# Modulation of β-Amyloid Fibril Formation in Alzheimer’s Disease by Microglia and Infection

**DOI:** 10.3389/fnmol.2020.609073

**Published:** 2020-11-26

**Authors:** Madeleine R. Brown, Sheena E. Radford, Eric W. Hewitt

**Affiliations:** School of Molecular and Cellular Biology and Astbury Centre for Structural Molecular Biology, Faculty of Biological Sciences, University of Leeds, Leeds, United Kingdom

**Keywords:** β-amyloid, Aβ, amyloid fibril, amyloid plaques, Alzheimer’s disease, infection, microglia

## Abstract

Amyloid plaques are a pathological hallmark of Alzheimer’s disease. The major component of these plaques are highly ordered amyloid fibrils formed by amyloid-β (Aβ) peptides. However, whilst Aβ amyloid fibril assembly has been subjected to detailed and extensive analysis *in vitro*, these studies may not reproduce how Aβ fibrils assemble in the brain. This is because the brain represents a highly complex and dynamic environment, and in Alzheimer’s disease multiple cofactors may affect the assembly of Aβ fibrils. Moreover, *in vivo* amyloid plaque formation will reflect the balance between the assembly of Aβ fibrils and their degradation. This review explores the roles of microglia as cofactors in Aβ aggregation and in the clearance of amyloid deposits. In addition, we discuss how infection may be an additional cofactor in Aβ fibril assembly by virtue of the antimicrobial properties of Aβ peptides. Crucially, by understanding the roles of microglia and infection in Aβ amyloid fibril assembly it may be possible to identify new therapeutic targets for Alzheimer’s disease.

## Introduction

**FIGURE 1:**
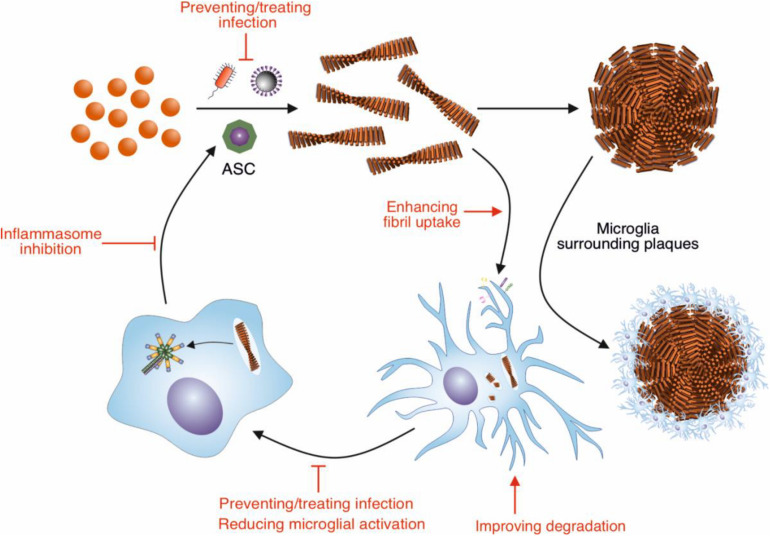
Modulation of Aβ amyloid fibril assembly and clearance by microglial cells and infection, and potential targets of intervention. Aβ peptide assembles into highly ordered amyloid fibrils with a characteristic cross-β structure. These fibrils form the core of amyloid plaques present in AD brains. Microglia surround these plaques, forming a protective barrier around them, limiting the recruitment of further Aβ. Microglia can also contribute to the clearance of Aβ fibrils. In order to remove Aβ deposits in AD, the phagocytic activity of microglia could be enhanced by targeting receptors and pathways involved in this response, such as TREM2 and CD33. Increasing the breakdown by microglial proteases could also enhance clearance of Aβ deposits. The NLRP3 inflammasome in microglia is concurrently activated in response to Aβ fibrils, resulting in the release of ASC specks. These specks cross-seed the formation of Aβ fibrils, resulting in further Aβ aggregation. Therefore, inhibiting the activation of the NLRP3 inflammasome would reduce the cross seeding of Aβ aggregation by ASC specks. Aβ also has antimicrobial and antiviral-properties, assembling into amyloid fibrils in response to infection. Thus, infection could be targeted to reduce Aβ aggregation.

Alzheimer’s disease (AD) is the most common form of dementia and is characterized by brain atrophy, amyloid plaques, intracellular neurofibrillary tangles, and neuroinflammation (Braak and Braak, 1991; Jack et al., 1998; Heppner et al., 2015). The amyloid plaques are primarily composed of fibrils formed by the β-amyloid (Aβ) peptides (Wang et al., 1996). In AD Aβ assembles into fibrils within the highly complex environment of the brain; as such multiple molecular and cellular factors may influence not only the formation the fibrils, but also their clearance. In contrast, Aβ fibril formation is typically studied *in vitro* by incubating the purified peptide in simple solution conditions. This may not reproduce how Aβ fibrils assemble in the AD brain, and result in the generation of fibrils that have different properties to those formed *in vivo.* Indeed, Aβ fibrils made *in vitro* do not efficiently induce amyloid plaque formation when injected into the hippocampus of young AD model mice, whereas brain extracts from AD patients and aged AD model mice lead to Aβ deposition into plaques (Meyer-Luehmann et al., 2006). This suggests that there are cofactors present *in vivo* that promote Aβ fibril assembly and deposition in AD. This review will focus on two potential cofactors, microglia and infection, and how these modulate Aβ amyloid fibril assembly and whether these can be targeted to reduce plaque formation (Figure 1).

## Aβ Amyloid Fibril Assembly

Aβ is formed by the sequential cleavage of transmembrane protein amyloid precursor protein (APP) by β-secretase and γ-secretase, resulting in Aβ fragments ranging from 39 to 43 residues in length (Selkoe, 1998). The predominant forms of the peptide are the 40- and 42- residue peptide variants Aβ_1–40_ and Aβ_1–42_ (Wang et al., 1996). All known dominant mutations associated with early-onset AD occur in APP or in presenilin-1 (PSEN1) and presenilin-2 (PSEN2), which are components of γ-secretase (Karch et al., 2014). Genome-wide association studies (GWAS) have also been used to identify genetic risk factors for late-onset AD, and this has identified genes that encode proteins involved in APP processing including SORL1, ADAM10, and APH1B (Lambert et al., 2013; Jansen et al., 2019). This genetic evidence implicates Aβ as an initiating factor in AD.

Aβ peptides are intrinsically disordered in their monomeric form and assemble into highly ordered fibrils via a nucleation dependent pathway, in which monomers self-associate to form a nucleus (Knowles et al., 2014). Addition of further Aβ peptides to the nucleus culminates in the formation of fibrils, which can then be elongated by end on addition of Aβ peptides. An array of oligomeric forms of Aβ are associated with fibril assembly reactions and many studies point to a key role for these oligomers in neurotoxicity (Shankar et al., 2008; Evangelisti et al., 2016; Serra-Batiste et al., 2016). In addition, in secondary nucleation, the surface of existing Aβ fibrils can catalyze the formation of new Aβ fibrils (Cohen et al., 2013). Cross-seeding can also occur in which other protein complexes, including fibrils of other amyloidogenic sequences, provide surfaces for the secondary nucleation of Aβ fibril assembly (Morales et al., 2013; Ono et al., 2014; Moreno-Gonzalez et al., 2017).

Aβ amyloid fibrils are highly ordered, with a common cross-β structure, consisting of β-sheets in which in-register β-strands are oriented perpendicularly to the fibril axis, with 4.6–4.7 Å spacing between them (Eanes and Glenner, 1968). Fibrils are unbranched, typically 5–15 nm in width, can reach up to several microns in length and can consist of a number of cross-β subunits (Iadanza et al., 2018). These subunits are protofilaments which associate to form a mature Aβ fibril (Iadanza et al., 2018). While all Aβ fibrils share this characteristic cross-β structure, polymorphism refers to the different molecular structure of the peptide within this cross-β subunit, and also the different number and arrangement of the cross-β subunits that make up a mature fibril (Tycko, 2015). While Aβ fibrils formed both *in vitro* and derived from *ex vivo* patient tissue exhibit polymorphism, the structures determined to date of fibrils derived from AD patient tissue are distinct from those formed *in vitro* (Petkova et al., 2005; Paravastu et al., 2008, 2009; Lu et al., 2013; Qiang et al., 2017; Kollmer et al., 2019). In addition, it was shown that synthetic Aβ fibrils do not efficiently induce Aβ-plaque formation when injected into the hippocampus of young AD model (APP23) mice, whereas brain extracts from AD patients and aged APP23 mice led to Aβ deposition (Meyer-Luehmann et al., 2006). This suggests that a cofactor, or multiple cofactors within the brain, could be required to drive Aβ assembly and deposition *in vivo* (Meyer-Luehmann et al., 2006).

## Microglia and the Immune Response to Aβ Amyloid Fibrils

Microglia are immune cells that are resident in the brain, and depending on the region, make up 0.5–16% of all cells in the human brain (Mittelbronn et al., 2001; Ajami et al., 2007; Ginhoux et al., 2010). When in a resting state, microglia have a ramified morphology, multiple fine processes project from the cell body, which are used to monitor the central nervous system (CNS) microenvironment (Nimmerjahn et al., 2005). These cells respond to changes in the local environment and migrate to activating stimuli, adopting a more amoeboid morphology and expressing an altered repertoire of receptors (Davalos et al., 2005). Reactive microglia are observed in AD brains, in close association with Aβ plaques (Itagaki et al., 1989; Yuan et al., 2016). Aβ fibrils have been shown to activate the production of pro-inflammatory cytokines by microglia and thus may be a stimulus for the increased production of these cytokines, which contribute to the neurodegeneration associated with AD (Griffin et al., 1989; Bauer et al., 1991; Patel et al., 2005; Halle et al., 2008; Ojala et al., 2009). However, in addition to cytokine production, microglia may also modulate the formation of Aβ amyloid fibrils and plaques. Crucially, microglia can affect both the generation and the degradation of Aβ fibrils. The balance between these activities may therefore represent a key determinant in whether amyloid plaques accumulate in the AD brain.

## Formation of Physical Barriers Around Aβ Amyloid Plaques by Microglia

In AD microglia migrate to, surround and infiltrate Aβ amyloid plaques, where they come into close contact with Aβ fibrils (Itagaki et al., 1989). In AD mice models this recruitment can occur as quickly as within a day of plaque formation and results in a two-fivefold increase in microglia at Aβ plaques compared to the neighboring tissue (Frautschy et al., 1998; Simard et al., 2006; Meyer-Luehmann et al., 2008). Microglia have been shown to surround plaques forming a barrier that limits their outward growth by preventing the recruitment of Aβ peptides (Condello et al., 2015). Plaques with less microglial coverage were less compact and had increased recruitment of soluble Aβ_1–42_, allowing the formation of Aβ_1–42_ protofibrils (Condello et al., 2015). Similar results were found after depletion of microglia with PLX5622, an inhibitor of the essential microglial colony stimulating factor 1 receptor (CSF1R) signaling pathway (Spangenberg et al., 2019). These hotspots of Aβ_1–42_ protofibrils were found to be neurotoxic, resulting in more severe neuritic dystrophy (Condello et al., 2015). This supports the role of microglia in the formation of a physical barrier around fibrillar plaques, compacting plaque cores, limiting growth and reducing neurite dystrophy (Condello et al., 2015).

An array of genes is associated with the development of late-onset AD (Karch et al., 2014; Jansen et al., 2019). One of these genes encodes triggering receptor expressed on myeloid cells 2 (TREM2), an immune receptor of the immunoglobulin family, which is expressed by microglia (Lambert et al., 2013). TREM2 sequence variants R47H and R62H have been found to increase the risk of developing late-onset AD (Jonsson et al., 2013; Chih Jin et al., 2014). This plasma membrane receptor forms signaling complexes with the adaptor protein DNAX-activating protein of 12 kDa (DAP12), and is important in the phagocytosis of apoptotic neurons and the negative regulation of inflammatory responses (Hamerman et al., 2005, 2006; Takahashi et al., 2005; Piccio et al., 2007; Hsieh et al., 2009). In AD model mice, the deletion of TREM2 did not significantly affect Aβ deposition, but it did reduce the extent to which microglia surrounded Aβ plaques (Ulrich et al., 2014; Wang et al., 2016). Plaques were more diffuse, and this was associated with an increased level of neuritic damage. This suggests a requirement for TREM2 in the formation of a neuroprotective microglial barrier. In support of this role for TREM2, Yuan et al. (2016) found that mice haplodeficient for TREM2 or DAP12 and humans harboring the R47H TREM2 mutation had a reduced microglial barrier surrounding Aβ plaques, and reduced plaque compaction. The Aβ fibrils in these plaques were found to be longer and there was more evidence of Aβ nanostructures extending out from the main Aβ fibril bundle, allowing more interaction with nearby neurites (Yuan et al., 2016).

## Clearance of Aβ by Microglia

In addition to surrounding amyloid plaques, the microglia recruited to these plaques may be involved in clearing these amyloid deposits (Rogers et al., 2002). Microglia are thought to contribute to the clearance of Aβ via the secretion of amyloid-degrading enzymes and by the internalization of Aβ fibrils. Furthermore, analysis of gene regulatory networks in late-onset AD identified that immune and microglial molecular networks were most associated with late-onset AD (Zhang et al., 2013). A number of these risk genes have been found to be involved in the clearance of Aβ (Kleinberger et al., 2014; Ulrich et al., 2018; Griciuc et al., 2019) highlighting the importance of this process in AD.

### Secreted Microglial Proteases

Enzymes that cleave Aβ include the metalloendopeptidases insulin-degrading enzyme (IDE) and neprilysin (NEP). Microglia are thought to contribute to the secretion of these enzymes, along with neurons and astrocytes, and a decrease in microglial expression of both enzymes is associated with aging in AD model mice (Leissring et al., 2003; Hickman et al., 2008; Tamboli et al., 2010). These enzymes, however, are thought to be limited to the degradation of monomeric peptide, and do not contribute to the degradation of Aβ amyloid fibrils (Qiu et al., 1998; Farris et al., 2003; Leissring et al., 2003). There is also evidence on the capability of NEP to degrade some oligomeric forms of Aβ. The enzyme was found to degrade oligomers formed from synthetic Aβ peptide, but in another study NEP did not degrade Aβ oligomers secreted from cells overexpressing APP (Kanemitsu et al., 2003; Leissring et al., 2003).

Secreted enzymes capable of cleaving fibrillar Aβ have, however, been identified. Metalloprotease-9 (MMP-9) is a zinc-dependent metalloprotease expressed by neurons, astrocytes, microglia and vascular cells in the brain (Vafadari et al., 2016). It was shown that incubation of Aβ_1–40_ and Aβ_1–42_ fibrils with MMP-9 leads to their degradation (Yan et al., 2006). Fibril fragments produced were analyzed using mass spectrometry and this revealed species corresponding to Aβ_1–20_ and Aβ_1–30_, suggesting Phe20-Ala21 and Ala30-Ile31 as cleavage sites (Yan et al., 2006). These sites must be accessible to MMP-9 in the fibril structure. MMP-9 was also found to degrade compact Aβ amyloid plaques in brain sections from AD model (APP/PS1) mice (Yan et al., 2006). MMP-2 is implicated in the degradation of soluble Aβ, with increased Aβ_1–40_ and Aβ_1–42_ identified in the soluble fraction of cortex and hippocampal brain samples of knock out MMP-2 mice compared to wild-type controls (Yin et al., 2006).

### Uptake and Degradation of Aβ Fibrils by Microglia

Consistent with a role in the clearance of Aβ, microglia express an array of receptors that facilitate the uptake of Aβ aggregates.

#### Toll-Like Receptors

One family of receptors involved in the immune response to Aβ amyloid are the Toll-like receptors (TLRs) a class of pattern recognition receptors that recognize conserved microbial structures (Kawasaki and Kawai, 2014). TLRs are type I integral membrane proteins which recognize ligands with their leucine-rich repeat (LRR)-containing ectodomains. RNA sequencing revealed that the expression of six TLR genes (1,2,4,5,6,8) is upregulated in the temporal cortex of AD patients when compared to control brains, likely resulting from increased microglial activation (Chakrabarty et al., 2018). A direct interaction was identified between Aβ fibrils and CD14, a TLR co-receptor previously shown to associated with the inflammatory response to fibrillar Aβ (Fassbender et al., 2004; Reed-Geaghan et al., 2009). This interaction was shown to facilitate the internalization of Aβ fibrils by microglia, at lower concentrations than that required for cell activation (Liu et al., 2005). This suggests that CD14 could be involved in the phagocytosis of Aβ fibrils at low concentrations, but increased Aβ levels in AD results in cellular activation. Consistent with a role in Aβ uptake, TLR4 deficiency in AD mouse models results in increased fibrillar and soluble Aβ deposition (Tahara et al., 2006). Conversely, stimulation of the murine microglial cell line BV-2 with TLR2 and TLR4 ligands significantly increased the internalization of Aβ *in vitro*, further implicating TLR receptors in Aβ uptake and clearance (Tahara et al., 2006; Song et al., 2011).

#### Scavenger Receptors

Another family of receptors found to be involved in the internalization of Aβ fibrils are the scavenger receptors (SRs), which are highly expressed by microglia (Christie et al., 1996; Wilkinson and El Khoury, 2012). It was found initially that class A SRs, characterized by an extracellular collagen-like domain, are involved in the binding to Aβ fibrils to microglial cells (El Khoury et al., 1996). It was then shown that coincubation of microglia with SR ligands such as acetyl-low density lipoprotein (Ac-LDL) reduced Aβ uptake, and CHO cells transfected with class A, or class B SR’s showed enhanced Aβ uptake, suggesting that SRs are important in the uptake and clearance of Aβ (Paresce et al., 1996). Further investigation using microglia that are deficient in SR-A1 confirmed the role of SR-A1 and also SR-B1 in binding Aβ fibrils, consistent with a role in the clearance of Aβ amyloid (Husemann et al., 2001). CD36 is a class B scavenger receptor identified to form a receptor complex with the α_6_β_1_-integrin and the integrin-associated protein CD47 in microglia. This complex was shown to mediate the binding of Aβ fibrils to microglial cells and the subsequent activation of intracellular signaling pathways (Bamberger et al., 2003). While initial studies reported that Aβ fibril binding to this complex is largely involved in the activation of an inflammatory response, it was also reported that the interaction of Aβ fibrils with this complex is involved in the phagocytic uptake of fibrils by microglia (Coraci et al., 2002; Moore et al., 2002; Bamberger et al., 2003; Koenigsknecht and Landreth, 2004).

#### TREM2

The deletion of TREM2 in primary microglia was shown to significantly reduce the phagocytosis of aggregated Aβ_1–42_ (Kleinberger et al., 2014). Similarly, TREM2 deficiency reduced the efficacy of antibody-targeted Aβ phagocytosis by microglia (Xiang et al., 2016). There is evidence for direct interactions between TREM2 and Aβ_1–42_ fibrils, although no difference in binding affinity was identified for TREM2 R47H and R62H variants that are associated with an increased risk of AD (Lessard et al., 2018). However, the internalization of monomeric Aβ was reduced with the expression of these TREM2 AD variants (Lessard et al., 2018). In another study, TREM2 was found to bind to Aβ oligomers with a similar affinity to previously described Aβ receptors, CD36 and receptor for advanced glycation end products (RAGE), and this interaction was compromised by R47H and R62H TREM2 mutations (Zhao et al., 2018). In this study, TREM2 deficiency had little effect on Aβ uptake but led to significantly reduced Aβ degradation once internalized by microglia (Zhao et al., 2018). In TREM2 knock out mice injected with Aβ oligomers, there was reduced microglial migration to the site of injection and reduced Aβ clearance (Zhao et al., 2018). A recent study found that loss of TREM2 function led to an acceleration in early amyloidogenesis, accompanied by a reduction in microglial recruitment as previously described, again suggesting that TREM2 has a role in microglial clearance of Aβ (Parhizkar et al., 2019). Together this evidence suggests that Aβ is a ligand for TREM2, and that TREM2 has a role to play in both Aβ clearance and Aβ-stimulated microglial activation.

### A Novel Role for the Autophagy Machinery in Aβ Receptor Recycling

Aβ clearance by microglia may involve proteins of the autophagy machinery in a pathway distinct from their canonical function (Heckmann et al., 2019). This pathway is referred to as LC3-associated endocytosis (LANDO), with LC3 being a key protein in macroautophagy. Evidence from this study suggests that LANDO facilitates recycling of the Aβ receptors CD36, TLR4 and TREM2, thus allowing cycles of Aβ endocytosis to continue, promoting Aβ uptake and clearance (Heckmann et al., 2019). The autophagy proteins ATG5 and Rubicon were found to be protective against Aβ deposition, with their absence leading to increased pathology. The expression of autophagy proteins declines with age, which may be related to the development of Aβ pathology in AD (Rubinsztein et al., 2011). It is important to note that macroautophagy has previously been implicated in the secretion of Aβ into the extracellular space where it forms plaques in AD (Nilsson et al., 2013). When autophagy-related gene 7 (ATG7) was conditionally knocked out in excitatory neurons of APP transgenic mice, extracellular Aβ plaque pathology was significantly decreased, and Aβ instead accumulated intracellularly (Nilsson et al., 2013). Thus, a reduction in expression of proteins involved in macroautophagy could affect both Aβ secretion and clearance.

### CD33

CD33, a type 1 transmembrane protein, is a sialic acid-binding immunoglobulin-like lectin (Siglec) expressed by immune cells, and was identified by GWAS to be associated with AD (Hollingworth et al., 2011). In addition, CD33-positive microglia and CD33 protein levels were found to be increased in AD brains, and CD33 was found to be associated with cognitive decline (Karch et al., 2012; Griciuc et al., 2013). It was found that a rs3865444 allele that was found to be protective in AD led to a reduction in the level of insoluble Aβ in the AD brain, suggesting a role for CD33 in mediating the clearance of Aβ (Griciuc et al., 2013). Furthermore, a risk allele of rs3865444 was associated with reduced Aβ_1–42_ internalization, and an increase in fibrillar amyloid, and neuritic amyloid pathology in AD patients, supporting the involvement of CD33 in the modulation of Aβ clearance (Bradshaw et al., 2013). Recent work by Griciuc et al. (2019) showed that knockout of CD33 led to mitigated Aβ pathology in 5xFAD AD model mice, with genes related to phagocytosis found to be upregulated (Griciuc et al., 2019). The opposite effects were found to result from TREM2 knockout (Griciuc et al., 2019). Interestingly, this differential gene expression in CD33 deficient 5xFAD mice only occurred in the presence of TREM2, suggesting that TREM2 acts downstream of CD33 (Griciuc et al., 2019).

### Degradation of Aβ Fibrils by Lysosomal Proteases

Once internalized Aβ fibrils are sorted to lysosomes, a degradative organelle which contains proteases that are capable of degrading Aβ fibrils (Paresce et al., 1997). Aβ_1–42_ monomeric peptide, non-fibrillar assemblies and fibrils were all shown to be cleaved by the lysosomal cysteine protease cathepsin B, resulting in the production of Aβ_1–40_, Aβ_1–38_ and Aβ_1–33_ in a dose-dependent manner (Mueller-Steiner et al., 2006). This suggests an anti-amyloidogenic role for cathepsin B, via the C-terminal truncation of Aβ. In addition to this, cathepsin B was found to accumulate in mature amyloid plaques in AD model mice. Cathepsin B activity was highest in supernatant taken from primary microglial cell cultures, compared to neurons and astrocytes, suggesting that these cells act as a source of cathepsin B as they surround Aβ plaques (Mueller-Steiner et al., 2006). The lysosomal protease, tripeptidyl peptidase 1 (TPP1) is another enzyme capable of cleaving Aβ fibrils. Digestion of Aβ_1–42_ fibrils *in vitro* by TPP1 revealed a number of different cleavage sites within the β-sheet domains, and molecular dynamics simulations demonstrated that these cleavages lead to destabilization of the β-sheet fibril structure (Solé-Domènech et al., 2018).

### Failure of Microglia to Clear Aβ Amyloid Fibrils in AD

Although evidence suggests that Aβ amyloid fibrils can be internalized by microglia and degraded by lysosomal and secreted proteases, microglia may be limited in their capacity to clear Aβ. This is evidenced by the accumulation of amyloid plaques in the AD brain despite microglial recruitment. A number of studies support this notion (Paresce et al., 1997; Majumdar et al., 2008). Fluorescently labeled Aβ fibrils internalized by cultured microglia were trafficked to lysosomes, however, Aβ was not degraded and was retained in microglial cells over a 3-day chase period (Majumdar et al., 2008). This was due to the inefficient delivery of chloride transporter CIC-9 to lysosomes, resulting in incomplete lysosome acidification and reduced activity of lysosomal proteases in the microglial (Majumdar et al., 2011).

Similarly, the genetic risk factor for AD, the ε4 allele of ApoE, may impair the ability of microglia to remove Aβ deposits. ApoE is a key cholesterol carrier, primarily produced by astrocytes in the brain, but also to some extent by microglia, and facilitates the transport of lipids via receptors of the low-density lipoprotein receptor (LDLR) family (Bu, 2009). Three common isoforms of ApoE exist in humans; ε2 ε3 and ε4 (Mahley, 1988). The ε4 allele of ApoE is the strongest genetic risk factor for late-onset AD, whereas the ε2 allele has a protective effect (Lambert et al., 2013). ApoE deletion in AD mouse models leads to reduced Aβ plaque deposition, implicating ApoE in Aβ amyloidogenesis and/or clearance (Bales et al., 1997; Ulrich et al., 2018). The efficiency of soluble Aβ clearance from the interstitial fluid of the brain is dependent on the ApoE isoform, with ApoE4 resulting in the least efficient clearance (Castellano et al., 2011). A number of mechanisms by which ApoE influences Aβ clearance have been proposed. In microglia, it was reported that lipidated forms of ApoE stimulate the degradation of soluble Aβ by NEP, with ApoE4 being the least efficient at promoting this degradation, and ApoE2 having the strongest effect (Jiang et al., 2008). There is also evidence to suggest that ApoE results in faster delivery of Aβ to lysosomes in microglia, by lowering cellular cholesterol levels, and the efficiency of this cholesterol efflux activity is isoform-dependent (Hara et al., 2003; Lee et al., 2011). Furthermore, microglial-like cells derived from human induced pluripotent stem cells expressing ApoE4 displayed reduced oligomeric Aβ_1–42_ phagocytosis compared to ApoE3 cells (Lin et al., 2018).

The ability of microglia to clear amyloid deposits in AD may also be diminished as a consequence of aging. Indeed, when production and clearance rates of Aβ_1–40_ and Aβ_1–42_ were tracked in AD patients using metabolic labeling, it was found that clearance rates for both peptides were reduced in AD compared to controls, but there were no differences in the rates of their production (Mawuenyega et al., 2010). This may be due to a reduced capacity of microglia to internalize Aβ fibrils. Microglia from older AD model mice have a twofold to sixfold reduction in expression of Aβ-binding receptors SR-A, CD36 and RAGE compared to wild type controls, in addition there was a significant reduction in the expression of secreted Aβ-degrading enzymes IDE, NEP and MMP-9 (Hickman et al., 2008). Old AD model mice were also found to have increased expression of inflammatory cytokines tumor necrosis factor-α (TNF-α) and interleukin-1β (IL-1β), indicating that while clearance pathways are impaired, a damaging inflammatory response to Aβ could be exacerbated (Hickman et al., 2008). A later study also found an impairment in phagocytic activity in AD mice compared to wild type controls, and this impairment correlated with an increased deposition of Aβ (Krabbe et al., 2013). Reducing Aβ load by administering an anti-Aβ antibody restored the phagocytic capacity of microglia, suggesting that the microglial dysfunction is a result of AD pathology (Krabbe et al., 2013).

A number of subset populations of microglia have been identified in aging and AD brains, with distinct transcriptional profiles and phenotypes (Keren-Shaul et al., 2017; Krasemann et al., 2017; Marschallinger et al., 2020). These include ‘damage associated microglia’ (DAM) which are proposed to play a protective role in disease (Keren-Shaul et al., 2017), and microglia with a neurodegenerative phenotype, which have lost their homeostatic function (Krasemann et al., 2017). The switch of microglia to this impaired neurodegenerative phenotype was found to be dependent on APOE signaling induced by TREM2, further implicating these pathways and microglial dysfunction in AD (Krasemann et al., 2017). Furthermore, a unique population was recently identified in the aging brain, termed ‘lipid-droplet-accumulating microglia’ (LDAM), which show a build-up of lipid droplets, and possess a distinct transcriptional signature (Marschallinger et al., 2020). Importantly, these microglia show defects in phagocytosis, as well as increased release of pro-inflammatory cytokines (Marschallinger et al., 2020). LDAM accounted for up to 50% of microglia in the hippocampus of aged mice, but have yet to be confirmed in AD models or brains (Marschallinger et al., 2020). Frigerio et al. (2019) showed that a microglial population termed ‘activated response microglia’ (ARMs) occur naturally in aging mice and in human brain, but the conversion to this state is accelerated in response to Aβ plaques (Frigerio et al., 2019). A number of AD risk genes including ApoE were found to be upregulated in ARMs, conversely depletion of ApoE blocked the recruitment of microglia to Aβ plaques (Frigerio et al., 2019). Given the association of the ApoE4 allele with AD, future studies should investigate whether this allele influences the production of ARMs (Lambert et al., 2013). Nonetheless, the implication of these data is that both aging and AD affect the phenotypes of microglia and thus their responses to Aβ.

## Clearance of Aβ by Astrocytes

In addition to microglia, astrocytes may play a role in the clearance of Aβ. Astrocytes, the most abundant glial cell in the brain, have numerous crucial roles in maintaining and regulating neuronal function and signal transmission (Perez-Nievas and Serrano-Pozo, 2018). Like microglia, astrocytes can react to pathogenesis by adopting a reactive phenotype, and this reactive astrogliosis is observed in AD brains, with a close relationship to Aβ pathology (Itagaki et al., 1989; Nagele et al., 2003; Perez-Nievas and Serrano-Pozo, 2018). Moreover, astrocytes may also contribute to the clearance of Aβ fibrils in AD and as discussed above the secreted protease MMP-9 is produced by a number of cell types including astrocytes (Vafadari et al., 2016). Astrocytes also secrete the MMP membrane type-1 (MT1) and kallikrein-related peptidase 7 (KLK7) (Liao and Van Nostrand, 2010; Kidana et al., 2018). MT1 is expressed by reactive astrocytes close to Aβ deposits and was shown to degrade Aβ plaques in an AD (APP) mouse model and cleave Aβ_1–42_ fibrils *in vitro* (Liao and Van Nostrand, 2010). KLK7 was found to cleave Aβ in the hydrophobic core motif of fibrils (KLVFFA), thus preventing fibril formation and promoting the degradation of pre-formed fibrils (Shropshire et al., 2014). KLK7 shows Aβ-degrading activity *in vitro*, and deletion of KLK7 in AD mice resulted in increased fibrillar Aβ pathology (Kidana et al., 2018). Further to this, KLK7 mRNA levels were found to be reduced in AD brains (Kidana et al., 2018).

A number of studies have also reported the ability of astrocytes to internalize Aβ, resulting in the accumulation of Aβ_1–42_ within activated astrocytes (Nagele et al., 2003; Wyss-Coray et al., 2003; Nielsen et al., 2010; Pihlaja et al., 2011). Cultured astrocytes were shown to migrate toward C-C motif ligand 2 (CCL2), a chemokine present at AD plaques, and subsequently bind to Aβ, although the receptors involved in in Aβ binding were not identified (Wyss-Coray et al., 2003). ApoE deficient astrocytes are, however, less efficient in the internalization and degradation of Aβ deposits compared to wild-type cells, thus implicating ApoE in astrocytic Aβ clearance (Koistinaho et al., 2004). Moreover, in a study of AD brain tissue, Aβ present within astrocytes was suggested to result from the phagocytosis of debris derived from damaged neurons, as neuron-specific markers were also identified (Nagele et al., 2003).

## Inflammasomes and the Cross Seeding of Aβ Aggregation

A role for activated microglia in the production of proinflammatory cytokines is well documented, and Aβ fibrils can act as a stimulus for this activation (Halle et al., 2008; Reed-Geaghan et al., 2009; Stewart et al., 2010). However, the pathway for the production of pro-inflammatory cytokines IL-1β and interleukin-18 (IL-18) in microglia may also promote Aβ fibril formation (Venegas et al., 2017). Thus, the relationship between microglia and Aβ is complex, and instead of attempting to remove amyloid plaques, microglia may also be playing a role in the formation of Aβ amyloid fibrils.

**FIGURE 2:**
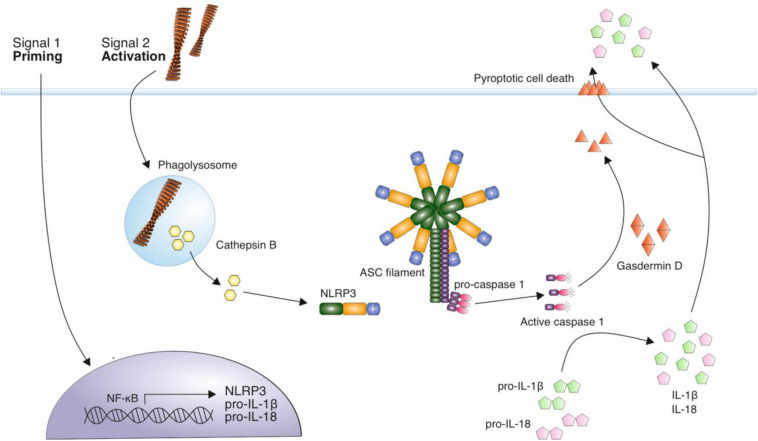
Mechanism of NLRP3 activation by amyloid fibrils. A priming signal, such as LPS, leads to the increased expression of NLRP3 and precursor forms of inflammatory cytokines IL-1β and IL-18. Aβ fibrils act as an activating stimulus via their internalization and disruption of lysosomes leading to the release of cathepsin B into the cytosol. This is thought to trigger the assembly of the NLRP3 inflammasome complex. This complex recruits ASC adaptor protein, which forms filaments. ASC filaments interact with pro-caspase 1 via CARD domains, resulting in caspase-1 activation. Active caspase 1 cleaves precursor forms of IL-1β and IL-18, which are secreted from immune cells in their active form, contributing to neuroinflammation. Caspase 1 also cleaves gasdermin D, which forms pores in the cell membrane, eventually resulting in pyroptotic cell death.

The NOD-like receptor family, pyrin domain containing 3 (NLRP3) inflammasome, is involved in the production and release of the pro-inflammatory cytokines IL-1β and IL-18 (Swanson et al., 2019). The activation of the inflammasome is a two-step process, requiring a priming stimulus followed by an activating stimulus. The priming stimulus can be cytokines, such as TNF-α and IL-1β, or pathogen associated molecular patterns (PAMPs) such as bacterial lipopolysaccharide (LPS) (Bauernfeind et al., 2009; Franchi et al., 2009). This priming step results in the transcriptional upregulation of inflammasome components, NALP3 and inactive forms of IL-1β, IL-18 and caspase-1 (Bauernfeind et al., 2009; Franchi et al., 2009). A number of stimuli can act as a second activating stimulus, including ATP, pore-forming toxins that result in low intracellular K^+^, crystalline structures such as uric acid and silica, and Aβ fibrils (Mariathasan et al., 2006; Martinon et al., 2006; Halle et al., 2008). The activating stimulus leads to the oligomerization of NLRP3, and the recruitment of adaptor protein apoptosis-associated speck-like protein containing a CARD (ASC). This triggers ASC polymerization into helical fibrils and subsequently assembly into micrometer-sized structures known as specks (Masumoto et al., 1999; Franklin et al., 2014; Lu et al., 2014). Caspase-1 is recruited via a caspase recruitment (CARD) domain and this results in caspase-1 autoproteolytic cleavage and activation. Caspase-1 is then responsible for the cleavage and thus activation of cytokines IL-1β and IL-18, which are released from cells, contributing to inflammation (Figure 2).

The activation of the inflammasome by Aβ fibrils was first shown *in vitro* and was dependent on Aβ phagocytosis and the subsequent damage to lysosomes, resulting in the release of cathepsin B into the cytosol (Halle et al., 2008). A further study then demonstrated that when NLRP3 or caspase-1 was knocked out in transgenic AD model mice, IL-1β activation was substantially reduced, providing support for the role of this activation pathway *in vivo* (Heneka et al., 2013). Furthermore, increased levels of cleaved caspase-1 were identified in AD patient brains compared with controls in hippocampal and cortical lysates, implicating the NLRP3 inflammasome as an important pathway in disease (Heneka et al., 2013).

**FIGURE 3:**
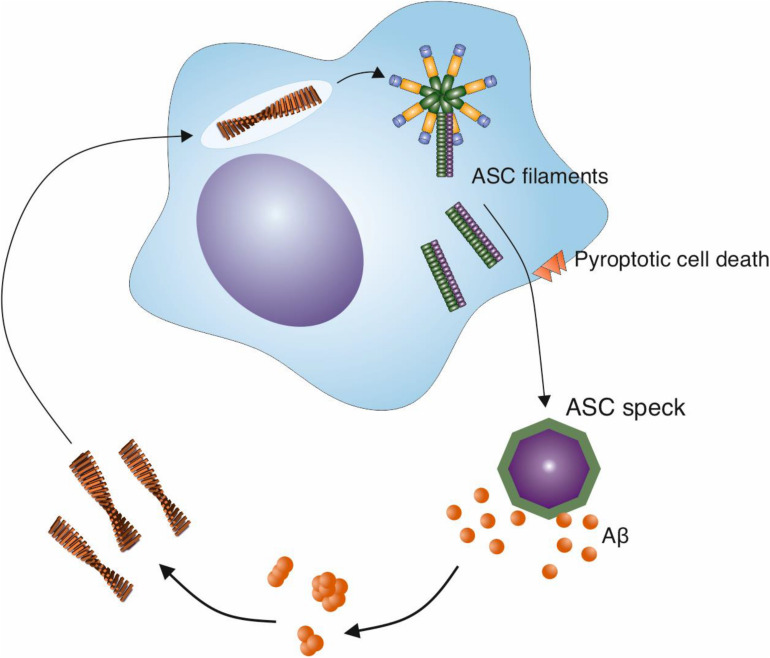
ASC specks released from activated microglia cross-seed Aβ aggregation. The NLRP3 inflammasome is activated in microglia in response to a variety of stimuli, including Aβ fibrils. This activation leads to the release of specks formed from ASC, an adaptor protein that is involved in the inflammasome pathway. ASC specks cross-seed Aβ peptide, resulting in the formation of further Aβ aggregates in the brain and may create a cycle of inflammasome activation and amyloid formation.

Crucially, in addition to the activation of the NLRP3 inflammasome by Aβ fibrils, evidence is emerging for a positive effect of the NLRP3 inflammasome on Aβ aggregation (Figure 3). The activation of the NLRP3 inflammasome results in the release of ASC specks (Swanson et al., 2019). Venegas et al. (2017) demonstrated that after their release, ASC specks bind to Aβ_1–42_ peptide (Venegas et al., 2017). *In vitro* experiments revealed that ASC specks accelerate the aggregation of both Aβ_1–40_ and Aβ_1–42_ into oligomers and protofibrils, indicating a cross-seeding activity (Venegas et al., 2017). This was dependent on the PYD domain of ASC. Moreover, when purified ASC specks were injected into the hippocampus of AD model mice, more Aβ deposits were observed, whereas an anti-ASC-speck antibody was capable of reducing Aβ deposition (Venegas et al., 2017). This suggests that not only do Aβ fibrils act as a stimulus to trigger microglial activation, but also that the result of this activation is the formation of Aβ aggregates, effectively producing a positive feedback loop (Figure 3). To compound this, in the presence of ASC-Aβ composites, consisting of Aβ oligomeric complexes forming in close association with ASC fibrils, the phagocytic clearance of Aβ by microglia was reduced by 35%, and its degradation was reduced (Friker et al., 2020). Thus, the activation of the NLRP3 inflammasome by Aβ fibrils could therefore both increase amyloid formation and reduce its clearance, contributing to the Aβ deposition observed in AD.

## Antimicrobial and Antiviral Properties of Aβ and Its Aggregation

### Aβ Response to Bacteria and Fungi

Infection may be a co-factor in the aggregation of Aβ into amyloid fibrils, moreover this may be related to the intrinsic antimicrobial activities of Aβ peptides. Indeed, it was found that Aβ inhibits the growth of eight common microorganisms including both bacterial and fungal species, at a similar potency to the *bone fide* antimicrobial peptide LL-37 (Soscia et al., 2010). A further study reported that Aβ protects against fungal and bacterial infections in mouse, *Caenorhabditis elegans* and cell culture models (Kumar et al., 2016). This was as a result of Aβ binding to microbial cell wall polysaccharides via its heparin-binding domain (VHHQKL) (Kumar et al., 2016). Aβ oligomers exhibited significantly increased binding compared to the monomeric peptide, and electron microscopy imaging revealed Aβ fibrillation, with fibrils associating with and linking together microbial cells into clumps, a process known as agglutination (Kumar et al., 2016). Another study found that this microbial agglutination was mediated by Aβ_1–42_, but not Aβ_1–40_, suggesting that the more amyloidogenic form of this peptide has greater antimicrobial activity (Spitzer et al., 2016).

The demonstration of the antimicrobial properties of Aβ suggests that AD may have an infectious etiology. A relationship has been proposed between gut microbiota and brain amyloidosis. APP AD model mice were found to have an altered gut microbiome compared to wild-type mice, and when APP mice were bred under sterile conditions, a significant reduction in cerebral Aβ pathology was observed (Harach et al., 2017). Furthermore, fecal transplants from APP mice bred in standard conditions to APP mice bred in sterile conditions resulted in increased Aβ pathology, whereas fecal transplants from wild-type mice did not have this effect (Harach et al., 2017). This research has been supported by studies in humans, with differences identified in the abundance of pro- and anti-inflammatory gut bacterial taxa in patients with brain amyloidosis (Cattaneo et al., 2017). When the bacterial taxonomic composition of fecal samples was compared between AD patient and control samples, a distinct microbiome composition was identified in AD samples (Vogt et al., 2017). Similarly, a number of studies have suggested a connection between the oral microbiome and AD (Ide et al., 2016; Chen et al., 2017; Dominy et al., 2019). *Porphyromonas gingivalis*, a pathogen in periodontal infections, was recently identified in AD brains and resulted in increased Aβ deposition in mice (Poole et al., 2013; Dominy et al., 2019).

### Aβ Response to Viruses

A number of studies have investigated the relationship between viral infections and AD. There is evidence that herpes simplex virus type-1 (HSV-1) is a risk factor for AD, when the AD patient is a carrier of the APOE-ε4 allele (Itzhaki et al., 1997). Subsequent studies have found an association between HSV-1 and the risk of neurodegenerative disease, with one retrospective cohort study in Taiwan identifying a 2.56-fold increased risk of dementia with HSV infection (Tzeng et al., 2018). However, another study reported only a slightly increased risk (Chen et al., 2018). In addition to HSV-1, analysis of genomic, transcriptomic, proteomic and histopathological data from brains identified increased human herpesvirus 6A (HHV-6A) and human herpesvirus 7 (HHV-7) in AD brain tissue samples compared to controls. Viral abundance was suggested to be linked with APP metabolism networks, including the induction of PSEN1 and BACE1 expression by HHV-6A (Readhead et al., 2018). However, the statistical robustness of this analysis was contested, and re-analysis of the data did not support a link between HHV-6A or HHV-7 and AD (Jeong and Liu, 2019).

Whilst the link between herpesvirus infections and AD may be equivocal, various studies suggest that herpesviruses and other infectious agents promote Aβ aggregation into amyloid fibrils. In neuronal and glial cell cultures, infection with HSV-1 led to the accumulation of Aβ within cells, and increased Aβ deposits were present in mouse brains after HSV-1 infection (Wozniak et al., 2007). Further investigation revealed that in AD, HSV-1 DNA localizes with Aβ plaques, with 90% of the plaques containing viral DNA (Wozniak et al., 2009). Similarly, it was shown that in a mouse model of recurrent HSV-1 infections, AD pathologies including Aβ accumulation, tau hyperphosphorylation and markers of neuroinflammation were observed (De Chiara et al., 2019). These results were corroborated recently in a 3D human brain-like model formed from human-induced neural stem cells (Cairns et al., 2020). It was found that whilst high HSV-1 infection levels led to cell death, low HSV-1 infection levels led to an AD-like phenotype, including dense Aβ fibrillar plaques and neuroinflammation (Cairns et al., 2020).

Both Aβ_1–40_ and Aβ_1–42_ inhibited the replication of HSV-1 in a number of cell lines when added to the cultures either prior to or in parallel with the virus (Bourgade et al., 2014). This effect was postulated to be a result of Aβ inserting into the HSV-1 envelope (Bourgade et al., 2014). Moreover, Aβ oligomers bind viral surface glycoproteins and fibrils mediate virus entrapment (Eimer et al., 2018). A recent study found that HSV-1 catalyzes the aggregation of Aβ_1–42_ *in vitro* and in an AD mouse model via surface-mediated nucleation, thus providing further support for this hypothesis (Ezzat et al., 2019). Crucially, the interaction of Aβ with viruses may be the same mechanism as its interaction with bacteria and fungi, namely the Aβ heparin-binding domain binds carbohydrates exposed on the surface of the virus (Eimer et al., 2018).

Not only does Aβ have antiviral activity, but its production may be controlled by the innate immunity protein, interferon-induced transmembrane protein 3 (IFITM3), which upregulates the activity of γ-secretase, resulting in the increased generation of Aβ peptide (Hur et al., 2020). Furthermore, deletion of IFITM3 in the 5xFAD mouse AD model resulted in reduced Aβ plaque formation, and IFITM3 expression was found to increase with aging and in mouse models expressing familial AD genes (Hur et al., 2020). IFITM3 plays a role in preventing viral infection, and its expression is induced by pro-inflammatory cytokines (Bailey et al., 2014). Thus, taken together these data support the antimicrobial and antiviral hypothesis for Aβ and suggest that infection may be a cofactor for Aβ aggregation *in vivo* (Jackson and Hewitt, 2017).

## How to Prevent Aβ Aggregation and Enhance Removal of Aβ Deposits

Aβ plaque formation in AD will be a balance between the rate of amyloid fibril assembly and the rate of clearance. Given their potential roles as cofactors in amyloid fibril assembly, targeting microglial activation and infections by viruses and bacteria may represent therapeutic approaches in AD. Similarly, enhancing the uptake and degradation of Aβ fibrils may provide an additional approach to reduce Aβ plaque burden in AD (Figure 1).

### Reducing the Activation of Microglia

Activated microglia are a characteristic feature of neuroinflammation in AD (Frautschy et al., 1998; Felsky et al., 2019). However, despite a decreased risk of AD associated with long-term non-steroidal anti-inflammatory drugs (NSAID) treatment, clinical trials of anti-inflammatory drugs to treat AD have not yet been successful (Aisen et al., 2003; Meyer et al., 2019; Howard et al., 2020). Minocycline, an anti-inflammatory tetracycline capable of crossing the blood-brain barrier (BBB), was found to reduce inflammatory markers and reverse cognitive impairment in an AD-like mouse model, induced by the administration of Aβ_1–42_ oligomers to the brain (Garcez et al., 2017). However, in clinical trials no improvement in cognitive impairment was identified with minocycline treatment (Howard et al., 2020). Similarly, treatment with naproxen did not slow disease progression in patients with mild-moderate, or reduce the progression of pre-symptomatic AD (Aisen et al., 2003; Meyer et al., 2019).

Whilst the aforementioned anti-inflammatories may be ineffective in the treatment of AD, the inflammasome may prove to be a better target. Indeed, a small molecule inhibitor of the NLRP3 inflammasome, MCC950, was found to stimulate Aβ phagocytosis *in vitro*, and reduce Aβ deposition in AD (APP/PS1) model mice (Dempsey et al., 2017). This was also associated with an improvement in cognitive function (Dempsey et al., 2017). Similarly, MCC950 prevented α-synuclein aggregate pathology and the degeneration of dopaminergic neurons in multiple rodent models of Parkinson’s disease (Gordon et al., 2018). These results are supported by a study in which NLRP3 components were knocked out in AD model mice, and this led to enhanced Aβ clearance and decreased Aβ deposition (Heneka et al., 2013). Importantly, these results support the clinical development of inflammasome inhibitors as a treatment for neurodegenerative amyloid diseases such as AD.

### Targeting Viral and Bacterial Infections

The demonstration that Aβ interacts with viruses may provide new routes of clinical intervention in AD patients; targeting viral infection could prevent the Aβ aggregation associated with AD. Two population cohort studies found that those taking anti-herpetic treatments for HSV infections had a reduced risk of dementia (Chen et al., 2018; Tzeng et al., 2018). In addition, in a recent study in 3D brain-like structures, HSV-1 infection induced an AD-like phenotype, and antiviral medication was successful in abrogating this phenotype, suggesting that antivirals could be utilized to treat AD patients (Cairns et al., 2020). An antiviral drug, Valacyclovir, is currently in Phase II clinical trials for the treatment of AD (ClinicalTrials.gov, ID# NCT03282916). Similarly, targeting bacterial infections could be used to prevent Aβ aggregation in AD. For example, inhibition of gingipains, toxic proteases from *P. gingivalis*, using small molecule inhibitors led to reduced Aβ_1–42_ production, neuroinflammation and neuronal death (Dominy et al., 2019). Consequently, a small molecule inhibitor of gingipains, COR388, is currently in Phase II clinical trials for the treatment of AD (ClinicalTrials.gov, ID# NCT03823404).

### Enhancing the Uptake and Degradation of Amyloid by Microglia

Whilst a role for TLR receptors in Aβ uptake by microglia has been suggested, these receptors also have a central role in the activation of inflammation (Reed-Geaghan et al., 2009; Stewart et al., 2010). Thus, targeting these receptors in the treatment of AD is not straight forward, as a detrimental inflammatory response could also be activated. Treatment with an LPS-derived TLR4 agonist, monophosphoryl lipid A (MPL) in a murine AD model led to reduced Aβ load and enhanced cognitive function, but a ‘low level’ inflammatory response was also triggered (Michaud et al., 2013). However, the AAV-mediated expression of the human TLR5 ectodomain as a ‘decoy’ receptor was explored, and found to result in the attenuation of Aβ plaque formation in a mouse model (Chakrabarty et al., 2018). The human TLR5 ectodomain was fused to human IgG4 Fc (sTLR5Fc), and this was found to bind to Aβ fibrils strongly, and to other forms of Aβ_1–40_ and Aβ_1–42_, to lesser extents. Therefore, the reduction in Aβ deposition into plaques could be due to the sequestration of fibrils by the TLR5 ectodomain (Chakrabarty et al., 2018). Furthermore, *in vitro* incubation with sTLR5Fc resulted in increased uptake of Aβ_1–40_ fibrils by microglia without activating TLR5 signaling (Chakrabarty et al., 2018), thus suggesting it as a safe method of immunomodulation in AD.

TREM2 is upregulated in response to increased Aβ levels in an AD mouse model (Jiang et al., 2014). Importantly, upregulating TREM2 significantly reduced Aβ deposition, neuroinflammation, synapse loss and led to improvements in cognitive function (Jiang et al., 2014). A monoclonal antibody targeting TREM2, AL002a, was found to activate TREM2 signaling *in vitro* (Price et al., 2020). Furthermore, treatment of 5xFAD mice with this antibody led to increased microglial recruitment to Aβ plaques, and reduced Aβ deposition (Price et al., 2020). TREM2 could therefore be a potential target for clinical intervention in the treatment of AD, and AL002 is currently being tested in Phase I clinical trials in patients with mild to moderate Alzheimer’s disease (ClinicalTrials.gov, ID#NCT03635047). Another monoclonal antibody, 4D9, was recently found to increase microglial uptake of Aβ *in vitro* and reduce Aβ deposits in the APP NL-G-F knock-in AD mouse model (Schlepckow et al., 2020). This antibody enhances TREM2 activity by competing for binding to the α-secretase cleavage site, therefore preventing TREM2 cleavage and subsequent shedding, whilst also enhancing TREM2 signaling (Schlepckow et al., 2020).

Macrophage colony stimulating factor (M-CSF) upregulates the transcription of the chloride transporter CIC-7 by microglia, increasing lysosomal acidification and enhancing the degradation of Aβ amyloid fibrils (Majumdar et al., 2011). This suggests that M-CSF could be used to promote Aβ amyloid clearance in AD. Indeed, in APP_(Swe)_/PS1 transgenic AD model mice M-CSF treatment resulted in a reduced number of Aβ deposits, a higher ratio of microglia with evidence of Aβ internalization, and reduced cognitive decline (Boissonneault et al., 2009). However, M-CSF is a hematopoietic cytokine that has been implicated in a number of inflammatory and autoimmune diseases and as a consequence M-CSF treatment could have deleterious inflammatory effects (Hamilton et al., 2016).

An alternative to enhancing pathways for fibril uptake and degradation is to inhibit negative regulators of these pathways. Evidence points toward a role for CD33 as a negative modulator of Aβ fibril clearance (Bradshaw et al., 2013; Griciuc et al., 2013, 2019), and inhibition of CD33 may represent a therapeutic strategy. A phase I clinical trial is underway for the monoclonal antibody, AL003, which targets and inhibits CD33 (ClinicalTrials.gov, NCT03822208). With evidence suggesting that CD33 deletion in mice reduces Aβ pathology and increases microglial expression of genes relating to phagocytosis, AL003 administration aims to inhibit CD33, thus increasing the clearance activity of microglia and reducing Aβ deposition (Griciuc et al., 2019). Similarly, CD22 could be targeted in AD. A recent study used CRISPR-Cas9 with RNA sequencing analysis to identify genes that are related to aging and lead to changes in microglial phagocytosis (Pluvinage et al., 2019). CD22 was identified as a receptor that negatively regulates phagocytosis and is upregulated in aged microglia. It was found that inhibiting CD22 with a CD22 blocking antibody improved the phagocytosis of Aβ oligomers and α-synuclein fibrils *in vivo*, supporting the hypothesis that AD results from age-related changes in microglia that reduce their amyloid clearing ability (Pluvinage et al., 2019). Similarly, manipulating microglia in order to favor a switch from dysfunctional phenotypes to a protective phenotype such as DAM could be a used as future approach to restore microglial function and enhance the clearance of Keren-Shaul et al. (2017); Krasemann et al. (2017), and Marschallinger et al. (2020).

## Discussion

Multiple different cofactors may influence Aβ assembly *in vivo*, including inflammation and infection. Moreover, the extent of amyloid plaques formation in AD will be dependent on the balance between Aβ fibril formation and the clearance and degradation of these deposits. Evidence points to microglia playing roles in both amyloid formation and its clearance (Lee and Landreth, 2010; Venegas et al., 2017), as such the balance between these microglial activities may be a factor in the accumulation of amyloid plaques. Yet, despite their recruitment to plaques, microglia do not appear to be able to halt the formation Aβ fibrils in AD. In the aging AD brain a reduction in the uptake and degradation of Aβ amyloid fibrils by microglia may cause these cells to be overwhelmed by the amyloid deposits (Hickman et al., 2008; Mawuenyega et al., 2010; Krabbe et al., 2013). Enhancement of the degradative activity of microglia therefore represent potential targets for therapeutic intervention in AD (Boissonneault et al., 2009; Majumdar et al., 2011).

Neuroinflammation is a damaging process in AD (Heppner et al., 2015), but via production of the inflammasome specks microglia could be exacerbating the disease by cross seeding Aβ amyloid formation (Venegas et al., 2017). Moreover, Aβ fibrils can themselves stimulate inflammasome formation and this raises the intriguing possibility that Aβ fibrils promote Aβ aggregation via microglia activation, resulting in a vicious cycle (Halle et al., 2008; Heneka et al., 2013; Venegas et al., 2017; Friker et al., 2020). As such the inflammasome may be a good target for AD therapeutics (Heneka et al., 2013; Dempsey et al., 2017; Gordon et al., 2018), as both inflammation and inflammasome-dependent Aβ fibril assembly could be reduced.

Whilst, Aβ aggregation has been thought of as being a pathological process, Aβ has properties consistent with it being an antimicrobial peptide (Soscia et al., 2010; Bourgade et al., 2014; Kumar et al., 2016; Spitzer et al., 2016; Hur et al., 2020). Indeed, there are a number of key similarities with the antimicrobial peptide LL-37, which can also assemble into amyloid fibrils (Sood et al., 2008; Jackson and Hewitt, 2017). Whilst a role for Aβ *in vivo* as an antimicrobial peptide is unclear, *in vitro* it can agglutinate viruses, bacteria and fungi by assembling into amyloid like structures on the surface of these infectious agents. This provides an additional mechanism by which Aβ assembly could be promoted *in vivo*, by virtue of its interaction with the surfaces of infectious agents (Kumar et al., 2016; Eimer et al., 2018; Ezzat et al., 2019). Moreover, infection would also be predicted to activate inflammation and could promote Aβ aggregation via the inflammasome (Swanson et al., 2019).

Although, bacteria, fungi, viruses and inflammasome specks can cross seed Aβ aggregation, little is known about the structure and properties of the fibrils produced. Crucially, both *in vitro* and *in vivo* Aβ fibrils can assemble into multiple different fibril polymorphs, in which the Aβ peptides have different arrangements in the fibril structure (Petkova et al., 2005; Paravastu et al., 2008, 2009; Lu et al., 2013; Colvin et al., 2015; Gremer et al., 2017; Kollmer et al., 2019). Little is known about the molecular structure of the Aβ aggregates produced by cross seeding by either specks and microorganisms *in vitro* nor how they relate to those formed *in vivo* in AD brain. This is important to know because Aβ fibril polymorphism *in vivo* is related to the type of AD presented (Lu et al., 2013; Qiang et al., 2017; Rasmussen et al., 2017). Similarly, whilst Aβ fibrils can be internalized and degraded by microglia, at least to some extent *in vitro* (Husemann et al., 2001; Tahara et al., 2006; Reed-Geaghan et al., 2009; Song et al., 2011) it is not known if polymorphism affects clearance of amyloid fibrils *in vivo*. It is plausible that Aβ fibril polymorphism could affect the affinity for microglial Aβ receptors and how the fibrils are degraded by microglial proteases. Thus, any fibril polymorphs that can escape microglial clearance may accumulate more in an AD brain.

In summary, *in vivo* multiple different cofactors, including microglia and infection, may influence the assembly of Aβ amyloid fibrils, and thus could represent targets for therapeutic intervention in AD.

## Author Contributions

MB, EH, and SR wrote and edited the manuscript. All authors contributed to the article and approved the submitted version.

## Conflict of Interest

The authors declare that the research was conducted in the absence of any commercial or financial relationships that could be construed as a potential conflict of interest.
